# Pneumothorax in a COVID-19 Patient Receiving Long-Term Mechanical Ventilation

**DOI:** 10.1590/0037-8682-0376-2021

**Published:** 2021-07-23

**Authors:** Erdem Yuzuak, Serdar Aslan, Ismet Mirac Cakir

**Affiliations:** 1 Giresun University, Faculty of Medicine, Department of Radiology, Giresun, Turkey.

A 58-year-old woman reported to the clinic while being followed up after a diagnosis of COVID-19. She was administered favipiravir, dexamethasone, moxifloxacin, and a carbapenem. On the sixth day of treatment, she was admitted to the intensive care unit and intubated due to an oxygen saturation of 80-85% with an oxygen mask and the development of dyspnea and tachypnea. She was followed up in the intensive care unit for a month while mechanical ventilation, and she had worsening respiratory acidosis and elevated airway pressure. The general condition of the patient deteriorated, and chest CT showed left pneumothorax. In addition, there were bronchial enlargements, consolidated areas, and pleural effusion accompanied by diffuse ground-glass opacity in the thoracic CT scan (Figure 1, arrow).

**FIGURE 1:**
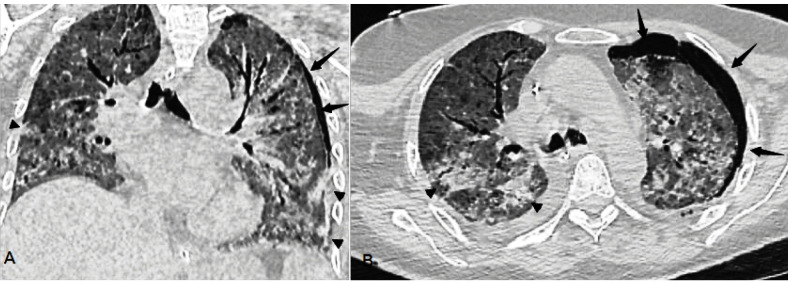
Axial and coronal unenhanced chest computed tomography. Pneumothorax (arrows), multiple ground-glass opacities and consolidations (arrowheads), in both lung fields.

Pneumothorax and pneumomediastinum are relatively common complications in patients with extensive alveolar damage requiring mechanical ventilation^1^. In lung areas exposed to high pressure during mechanical ventilation, alveolar rupture may develop and cause pneumothorax^2^. Cases of spontaneous pneumothorax have also been reported in patients who are not intubated with COVID-19^3^. According to one hypothesis, cystic and fibrotic changes in the lung parenchyma during the early stages of COVID-19 may be the cause of the predisposition to pneumothorax^3^.

## References

[1] Belletti A, Palumbo D, Zangrillo A, Fominskiy EV, Franchini S, Dell'Acqua A (2021). COVIDBioB Study Group. Predictors of
Pneumothorax/Pneumomediastinum in Mechanically Ventilated COVID-19
Patients. J Cardiothorac Vasc Anesth.

[2] Mohammadi Tofigh A, Shojaei SP, Zebarjadi Bagherpour J, Mirkheshti A, Tahmasebi H (2020). Pneumothorax as an Ominous Side Effect in COVID-19 Patients under
Mechanical Ventilation: Report of Seven Patients. J Cell Mol Anesth.

[3] Martinelli AW, Ingle T, Newman J, Nadeem I, Jackson K, Lane ND (2020). COVID-19 and pneumothorax: a multicentre retrospective case
series. Eur Respir J.

